# Determinants of genetic diversity in Neotropical salamanders (Plethodontidae: Bolitoglossini)

**DOI:** 10.1002/ece3.10707

**Published:** 2023-11-16

**Authors:** María Guadalupe Segovia‐Ramírez, Obed Ramírez‐Sánchez, Louis Paul Decena Segarra, Hairo Rios‐Carlos, Sean M. Rovito

**Affiliations:** ^1^ Unidad de Genómica Avanzada Centro de Investigación y de Estudios Avanzados del Instituto Politécnico Nacional Irapuato Mexico

**Keywords:** ddRADseq, evolutionary speed hypothesis, genetic variation, genome size, population size, transcriptomes

## Abstract

Genetic diversity is the raw material of evolution, yet the reasons why it varies among species remain poorly understood. While studies at deeper phylogenetic scales point to the influence of life history traits on genetic diversity, it appears to be more affected by population size but less predictable at shallower scales. We used proxies for population size, mutation rate, direct selection, and linked selection to test factors affecting genetic diversity within a diverse assemblage of Neotropical salamanders, which vary widely for these traits. We estimated genetic diversity of noncoding loci using ddRADseq and coding loci using RNAseq for an assemblage of Neotropical salamanders distributed from northern Mexico to Costa Rica. Using ddRADseq loci, we found no significant association with genetic diversity, while for RNAseq data we found that environmental heterogeneity and proxies of population size predict a substantial portion of the variance in genetic diversity across species. Our results indicate that diversity of coding loci may be more predictable than that of noncoding loci, which appears to be mostly unpredictable at shallower phylogenetic scales. Our results suggest that coding loci may be more appropriate for genetic diversity estimates used in conservation planning because of the lack of any association between the variables we used and genetic diversity of noncoding loci.

## Graphical abstract

Genetic diversity is the raw material of evolution but varies between species in ways that are not yet fully understood. We used two genomic datasets for Neotropical salamanders to show that while diversity of coding loci is well predicted by population size and environmental heterogeneity, diversity of noncoding loci may be largely unpredictable.

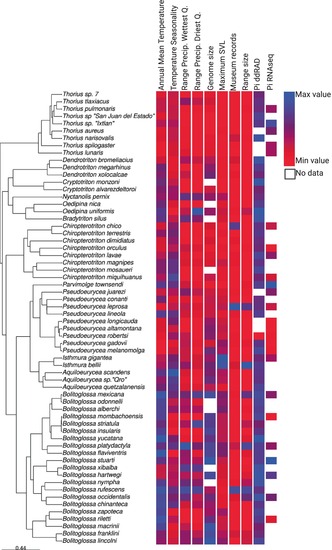

## 1 INTRODUCTION

Genetic diversity within populations is the raw material upon which microevolutionary forces act. Understanding how intraspecific genetic variation differs across species is essential to predict the ability of populations to adapt to novel environmental conditions; genetic divergence plays a key role in most models of speciation and is increasingly being incorporated into conservation planning (Coyne & Orr, 2004; Kardos et al., 2021). In particular, species' responses to ongoing climate change may be determined in part by how much genetic variation they possess at coding loci under selection or in regulatory regions. Despite its importance to both evolutionary studies and real‐world applications, our knowledge of the distribution of intraspecific genetic variation across the tree of life remains limited, as does our knowledge of the factors that cause variation in genetic diversity between species.

Following the discovery of high levels of genetic polymorphism within many populations (Lewontin & Hubby, 1966), numerous attempts have been made to explain levels of intrapopulation and intraspecific genetic variation (Ellegren & Galtier, 2016; Leffler et al., 2012; Romiguier et al., 2014; Singhal et al., 2017). While only mutation can generate novel genetic variation, both genetic drift and selection affect levels of standing variation within species. The strength of genetic drift depends on effective population size (N_e_), which can vary as a result of both species‐specific parameters (variance in reproductive output and sex ratios) and historical demography (Charlesworth, 2009). The neutral theory of molecular evolution (Kimura, 1968, 1983) predicts a positive relationship of diversity with both population size and mutation rate (with population‐level diversity for diploid species defined as $\theta =4N_{e}\mu$) and the nearly neutral theory predicts additional diversity in smaller populations due to the balance of selection and drift maintaining slightly deleterious mutations (Ohta, 1973). Selection can decrease genetic diversity directly by eliminating deleterious mutations or driving advantageous mutations to fixation as well as indirectly through selective sweeps on novel mutations (“genetic draft”) and background selection, both of which decrease diversity at linked sites (Charlesworth et al., 1993; Gillespie, 2000; Maynard Smith & Haigh, 1974). Thus, a comprehensive explanation for genetic diversity within populations and species would account for mutation rate, drift, direct selection, and selection at linked sites across the genome.

Multiple attempts to explain genetic diversity within species have been done at a broad phylogenetic scale, where attributes of morphology and life history vary widely. In a broad survey of intraspecific genetic diversity across animal phyla, various aspects of life history strategy (including propagule size and fecundity) predicted a large proportion of variance in genetic diversity while attributes of geographic range did not (Romiguier et al., 2014). A survey of nuclear genetic diversity within populations across animals, plants, and fungi found higher diversity in phyla primarily inhabiting aquatic environments, a correlation between range size and diversity in *Drosophila*, and an association of reproductive mode (selfing vs. outcrossing) and genetic diversity for plants (Leffler et al., 2012).

At shallower phylogenetic scales and between more ecologically similar species, however, diversity appears to be less predictable. In species of six families of European butterflies, body size and chromosome number (a proxy for the map length of the genome) were significant predictors of genetic diversity of transcriptomic loci, while life history attributes and an estimate of current population size were not (Mackintosh et al., 2019). A study in lizards of the genus *Ctenotus* showed a wide range of genetic diversity but only weak associations between genetic diversity and proxies of effective population size (Singhal et al., 2017). Within populations of *Ctenotus*, only the number of museum records per species explained genetic diversity of populations, while range size, number of museum records, and historical stability of range predicted intraspecific genetic diversity. The generation of data for additional groups is essential to improve our understanding of predictors of genetic diversity (Leffler et al., 2012).

An additional level of complexity is added when considering the impact of linked selection. Relatively few studies have examined the impact of linked selection on genetic diversity, in part because such studies require linkage maps that are not available for most species (Buffalo, 2021). The effect of linked selection should decrease with increasing recombination rate, as recombination events decrease linkage disequilibrium between loci on a chromosome. Genome size is inversely correlated with recombination rate per bp, and eukaryotic species with the largest genomes have estimated recombination rates per bp several orders of magnitude lower than those with the smallest genomes (Lynch, 2006). In a study using genomes with linkage maps for 40 animal and plant species, Corbett‐Detig et al. (2015) found a stronger correlation between levels of neutral genetic diversity and recombination rate for species with larger population sizes, providing support for the idea that natural selection removes more genetic variation in species with large population sizes. Their results suggest that the removal of genetic variation through natural selection at linked sites may provide an important part of the explanation of why levels of genetic diversity are constrained compared to neutral expectations. Coop (2016), however, showed that the magnitude of the effect of linked selection in their results is insufficient to explain the discrepancy between observed variation in genetic diversity among species and that expected from the neutral theory. Buffalo (2021) reanalyzed available data for 172 metazoan species and also found that linked selection is unlikely to explain the relationship between genetic diversity and census population size across species. Finally, a study in *Ficedula* flycatchers showed a positive association between chromosome length (which should be correlated with recombination rate per bp) and genetic diversity, possibly as a result of a lower density of targets of selection on larger chromosomes (Dutoit et al., 2017). Thus, additional work must be done in order to understand the relationship between chromosome size/recombination rate and genetic diversity as well as the underlying cause of this relationship.

Although a complete explanation for genetic diversity within species would necessarily involve predictors of variation in mutation rate, the factors affecting the mutation rate are myriad and relatively poorly understood. Mutation rate can vary between lineages based on differences in generation time, metabolic rate, population density, and efficiency of DNA repair (Baer et al., 2007; Krašovec et al., 2017). Multiple studies have shown a positive effect of temperature on mutation rate and reactive oxygen species formation (Muller, 1928; Oppold et al., 2016; Waldvogel & Pfenninger, 2021). In ectotherms, higher temperatures can increase mutation rate both directly, as a result of increased metabolic rate, and indirectly as a result of faster generation time. This increase of mutation rate with temperature, termed the Evolutionary Speed Hypothesis (Rensch, 1959; Rohde, 1992), was supported by analysis of mtDNA substitution rate in a global amphibian dataset (Wright et al., 2010). The Evolutionary Speed Hypothesis should apply across ectotherms, regardless of the particular natural history or life history of a group of organisms and thus represents an important potential source of genetic diversity within species.

Mixed results on the importance of factors controlling genetic diversity at broad scales as well as the apparent unpredictability of genetic diversity at shallower phylogenetic scales highlight the need for additional estimates of genetic diversity and analysis of patterns in more groups. Many of the links between genetic diversity and conservation are based on either noncoding nuclear loci or mtDNA, with coding nuclear loci less often included despite their likely higher relevance to species' ability to adapt to environmental changes. Here, we use proxies for the possible forces affecting genetic diversity within species (mutation, drift, direct selection, and linked selection) to understand which processes most strongly affect genetic diversity among species. We use an assemblage of Neotropical salamander species (family Plethodontidae, tribe Bolitoglossini; Wake, 2012) that show wide variation in proxies for census population size, potential selective pressures imposed by the external environment, mutation rate, and recombination rate. We use both transcriptomic loci that are expected to be under some level of selection and ddRAD loci that are likely noncoding to test the relative importance of drift, mutation rate variation, direct selection, and linked selection on coding and noncoding portions of the genome. By combining factors that have previously been tested separately for their effect on genetic diversity within species, our results contribute to our growing understanding of the forces that shape the raw material of evolution at microevolutionary scales.

## 2 METHODS

### 2.1 Sampling and generation of sequence data

**FIGURE 1:**
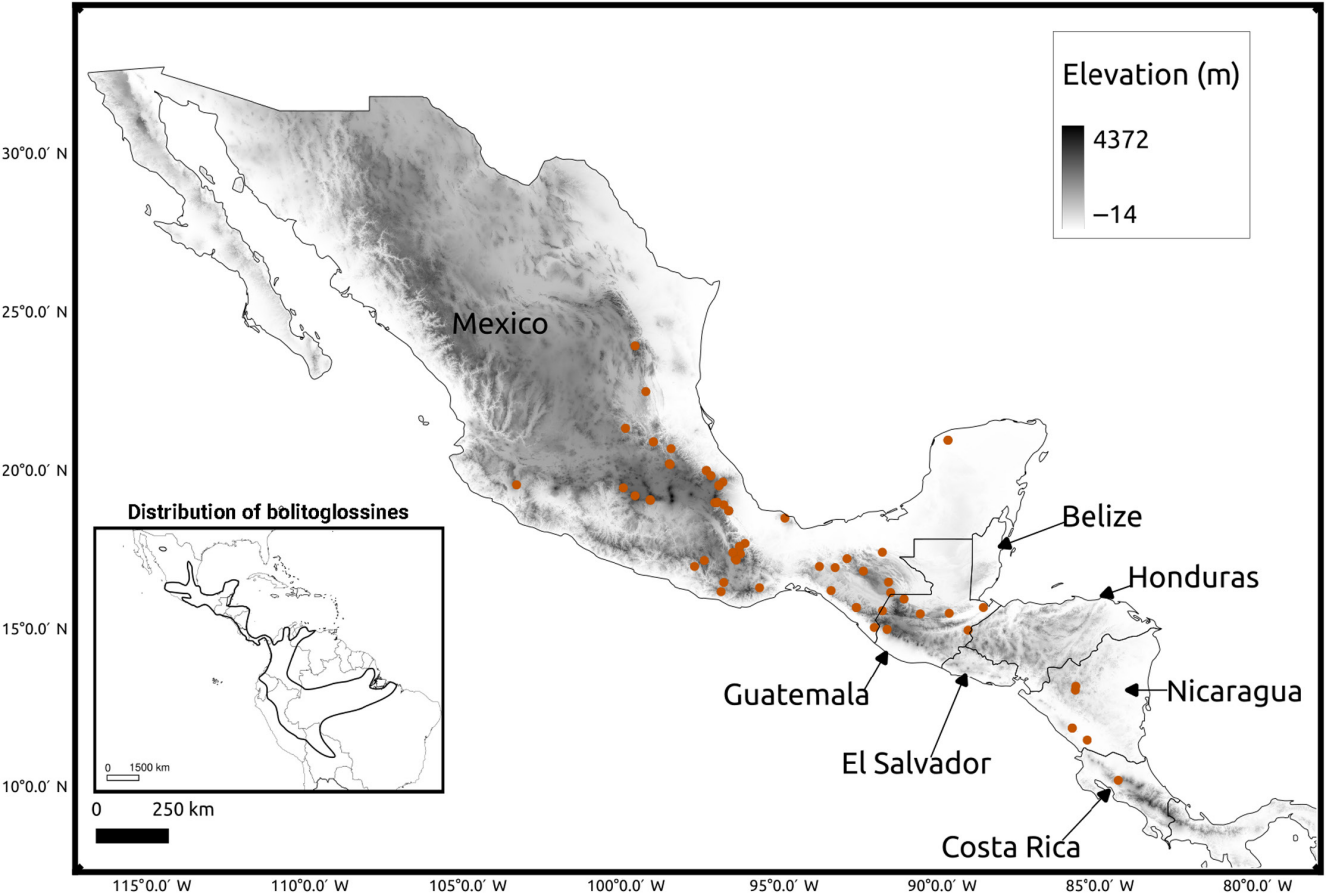
Map of samples used for genetic diversity estimation of bolitoglossine salamander species. Each point represents a single species included in the study.

We obtained tissues for 62 species of bolitoglossine salamanders from fieldwork and museum collections (Figure 1, Appendix 1). Most tissues were stored in NAP buffer (Camacho‐Sanchez et al., 2013), while some tissues from the Museum of Vertebrate Zoology (MVZ) were flash‐frozen and stored at −80°C or in liquid nitrogen. We decided to generate sequence data for a single individual per species in order to maximize the number of species included in the study, particularly because the large genome size of all species in the study requires higher than normal sequencing effort per sample. Singhal et al. (2017) found a high correlation between within‐species genetic diversity and within‐population genetic diversity (the latter estimated using a single individual per population). Furthermore, Dutoit et al. (2017) found nearly identical levels of genetic diversity across individuals of *Ficedula* flycatchers and suggested that genetic diversity estimates benefit more from increasing the number of loci than from including more individuals. Thus, using only one individual per species should be a reasonable proxy for within‐species genetic diversity.

We extracted DNA from liver tissue using the protocol of Aljanabi and Martinez (1997). We generated data for two different types of loci to test the effect of selection on genetic diversity: coding loci from transcriptomes and ddRAD loci, the large majority of which should be noncoding given that they are randomly distributed across the genome. We used a modified protocol for Illumina TruSeq RNA library Prep v2 (Illumina) for transcriptome libraries. In brief, modifications consisted in performing half‐reactions for all steps in the protocol to minimize the amount of RNA we needed for library construction because of limited tissue availability for some species of miniature salamanders. We prepared libraries for 25 species of salamanders (Appendix 1), six of which were sequenced as part of a previous project (Segovia‐Ramírez, 2017). These libraries from the previous project were sequenced on an Illumina HiSeq 4000 2 × 100 bp, while all libraries prepared specifically for this study were sequenced on one lane of an Illumina NovaSeq 6000 150 PE at the QB3 Vincent J Coates Genomics Sequencing Lab, University of California, Berkeley.

For ddRAD library construction, we followed a modified protocol based on Jones and Weisrock (2018), which was previously optimized for salamanders with large genomes. We used equal amounts of the enzymes EcoR1 and SphI for double digestion and indexed each library with unique P5 and P7 adaptors. Key adapted steps for salamanders with large genomes were the double digestion of 2–3 μg of gDNA (depending on genome size, with more DNA used for larger genomes) with SpHI and EcoR1 100KU (0.2 μL each one) in a total reaction volume of 50 μL for 6 h. The next key step was the first adaptor ligation with P1 and P2 of at least 1 μg of ddDNA to make pools before P5 and P7 indexing. Single‐stranded oligos were annealed to produce double‐stranded P1 and P2 adaptors before each ligation step. We performed a size selection step to select fragments from 300 to 500 bp prior to final amplification using a PippinPrep (Sage Biosciences). All libraries were sequenced at QB3 Vincent J. Coates Genomics Sequencing Lab, University of California, Berkeley (QB3 Genomics, RRID:SCR_022170) on an Illumina NovaSeq SP 2 × 150 bp.

### 2.2 Assembly, SNP calling, and genetic diversity estimation

For all RNAseq libraries, we followed a pipeline modified by Singhal (2013). This pipeline cleans raw reads by removing adapters, ambiguous base calls, low‐quality reads, contamination, and all unpaired reads using fastqc, cope, cutadapt, FLASH, trimmomatic, and bowtie2 (Andrews, 2010; Bolger et al., 2014; Langmead & Salzberg, 2012; Liu et al., 2012; Magoc & Salzberg, 2011; Martin, 2011). After cleaning, remaining reads were assembled de novo with Trinity v.2.2.0 (Grabherr et al., 2011). All assembled transcriptomes were annotated using four references from Ensembl (Cunningham et al., 2022): *Danio rerio*, *Xenopus laevis*, *Anolis carolinensis*, and *Homo sapiens*. We used only annotated transcripts for SNP calling and genetic diversity estimation to avoid inclusion of paralogous loci and transposable elements. For annotated transcripts, we chose the transcriptome with the most annotated loci (11,132 loci, *Pseudoeurycea leprosa*) as the reference for mapping with bwa‐mem (Li & Durbin, 2009) and as the reference and ancestor for SNP calling.

For the ddRAD data, we used the Stacks2 (Catchen et al., 2013) pipeline for cleaning and assembly. We cleaned and demultiplexed libraries with *process_radtags* and performed de novo assembly using *ustacks*. We did not annotate these ddRAD loci because of a lack of a plethodontid reference genome and because, given the large genome size and highly repetitive nature of plethodontid salamander genomes (Sun, López Arriaza, et al., 2012), most ddRAD loci are likely to fall outside coding regions. We assembled each species separately and used Bowtie2‐mem to map reads prior to SNP calling with ANGSD v. 0.937 (Korneliussen et al., 2014). We used *Bolitoglossa franklini* as the reference and ancestor for mapping and SNP calling (898,951 loci). Because of differences in median read depth between datasets, we called SNPs at sites with a read depth of at least six for ddRAD and 60 for transcriptomes using a probability threshold of 1e‐06 for both types of loci.

Because of the large size of the salamander genomes in our dataset and resulting reduction in sequence depth per locus, it is possible that we failed to obtain sequence data for both alleles for some ddRAD loci. Considering that low depth in ddRAD libraries can cause only one of the two alleles to be sequenced, we decided to estimate genetic diversity only from loci with at least one heterozygous site. To generate comparable estimates with transcriptomic data, we estimated genetic diversity from only heterozygous RNASeq loci. We estimated nucleotide diversity (*π*) for both ddRAD and transcriptome loci for each species by dividing the number of variable sites between two alleles at each locus by the length of the locus. We then calculated median nucleotide diversity for each species across all loci as our estimator of genetic diversity. Finally, we examined correlations of the number of polymorphic loci, genome size, and the number of species in the pool with mean coverage for each species in our ddRAD dataset in order to understand possible technical artifacts related to coverage differences between species that may have affected our results.

### 2.3 Predictors of genetic diversity

We included three proxies of census population size that have been used in prior studies of genetic diversity (Corbett‐Detig et al., 2015; Singhal et al., 2017). Body size (measured as Snout‐Vent Length [SVL]) should be inversely correlated with population size, as larger organisms tend to have lower population density. Geographic range size and the number of museum records should be positively correlated with population size. Singhal et al. (2017) found the number of museum records to be the proxy for population size with the highest explanatory power for predicting genetic diversity. We used the dataset of Decena‐Segarra et al. (2020) for maximum SVL for each species, complementing it with measurements of specimens and values from the literature for species not included in their dataset. We obtained museum records from the Global Biodiversity Information Facility (GBIF, www.gbif.org), using only points with voucher specimens. We checked all points against range maps to eliminate misgeoreferenced specimens or incorrect taxonomic identifications. After quality control, we retained over 30,000 records for the species in our dataset, some of which included more than one specimen. We used the total number of vouchered specimens for each species after quality control as a proxy for population size. We estimated geographic range size by constructing a 10 km buffer around each museum record and merging overlapping buffers for a species. We then summed the total area of all buffered points as an estimate of geographic range size. Values for proxies of population size are given in Appendix 2.

Although our ecological knowledge of most species of bolitoglossine salamanders is limited, a study of temperature selection and thermal variation in their environments showed that while most species select specific temperature ranges, they are often limited by the diversity of thermal environments with sufficient moisture (Feder, 1982). The ability of plethodontid salamanders to forage is largely limited by the rate of water loss, which depends on body size and relative humidity (Spotila, 1972). Thus, temperature and humidity are likely important constraints on multiple aspects of salamander life history, and both should impose selective constraints on salamanders. We used the range of two environmental variables as well as a point estimate of temperature seasonality to estimate environmental heterogeneity as a proxy for the strength of environmentally driven direct selection. We used the set of museum records for each species to extract the values of three variables from the WorldClim dataset (Hijmans et al., 2005) at a resolution of 30 arc‐sec (approximately 1 km^2^): Bio4 (temperature seasonality), Bio16 (precipitation of the wettest quarter), and Bio17 (precipitation of the driest quarter). We calculated the range of the two precipitation variables for each species by subtracting the minimum and maximum values recorded for the species. For microendemic species known from a single site, the range of climatic variables was equal to zero; in these cases, we changed zeros to a very small number (0.001) to avoid undefined values after log transformation of the data. For temperature seasonality, we used the value extracted from the locality used in our molecular dataset.

Because we have no estimate of mutation rate for the species in our study, we used mean annual temperature as a proxy for mutation rate following Wright et al. (2010), who showed that substitution rate varies with temperature in a global amphibian dataset. We extracted values for mean annual temperature (Bio1) from each voucher that we included in our sequencing dataset in order to have a measure of temperature at the exact locality from which we obtained sequence data. Values of environmental heterogeneity and mutation rate proxies are given in Appendix 2.

Recombination rate per base pair depends on both the physical length of chromosomes and the number of chiasmata that occur during meiosis. At least one chiasma must occur between homologous chromosomes for proper segregation during meiosis, and in many groups the number of chiasmata per chromosome or chromosome arm appears to be roughly constant; the number of chiasmata is proportional to the number of chromosome arms in humans and a survey of multiple frog families found 1–2 chiasmata per bivalent depending on chromosome size (Morescalchi & Galgano, 1973; Pardo‐Manuel de Villena & Sapienza, 2001). For a given number of chiasmata per chromosome, recombination rate per base pair across the genome should decrease with increasing chromosome size, leading to a reduction in genetic diversity as a result of linked selection. Because of the enormous genome size of salamanders, few linkage maps are available for the group; those that are available show wide variation in map size (Niedzicka et al., 2017; Smith et al., 2005; Voss et al., 2001). In multiple species of salamanders, approximately one chiasma per chromosome arm has been observed (reviewed in Niedzicka et al., 2017) and a linkage map of *Lissotriton vulgaris* (Salamandridae) was markedly shorter than the long maps found in *Ambystoma mexicanum* (Ambystomatidae) (which also shows an elevated number of chiasmata) and *Notophthalmus viridescens* (Salamandridae). Plethodontid salamanders, which show the widest variation in genome size within the salamanders, may have as many as three chiasmata per chromosome arm (Macgregor, 1993), but no linkage maps or published estimates of the number of chiasmata are available for Neotropical salamanders.

A major advantage of this group is that all members have 13 pairs of chromosomes (2N = 26), with homologous chromosomes essentially being scaled versions of each other across species depending on genome size (Sessions, 2008). Thus, if the number of chiasmata is relatively constant within the group, recombination rate per bp should decrease linearly with genome size, providing a relative index of the strength of linked selection across species. We use estimates of genome size from Decena‐Segarra et al. (2020) as well as new measurements obtained from Feulgen Image Analysis Densitometry (FIAD) as a predictor of recombination rate, with the caveat that variation in recombination rate across taxa could potentially make this a poor predictor of actual recombination rate. Genome size values for all species in our dataset are given in Appendix 2.

### 2.4 Model testing for predictors of genetic diversity

**FIGURE 2:**
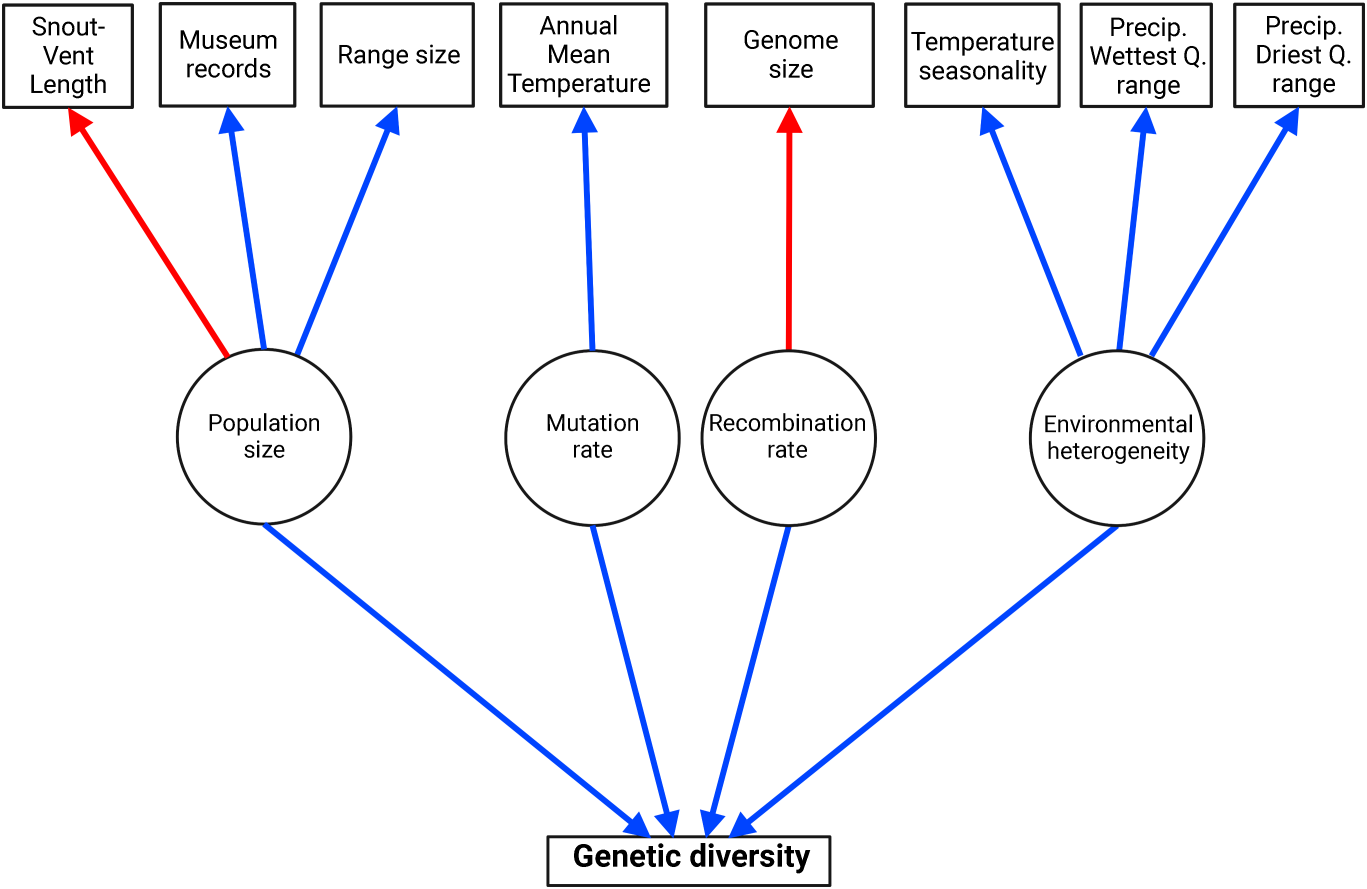
Diagram showing the hypothesized relationships between predictor variables, the evolutionary forces for which they are proxies, and genetic diversity. Squares indicate observed variables while circles show observed variables/processes measured by proxies. Blue arrows represent relationships hypothesized to be positive, and red arrows represent relationships hypothesized to be negative.

We hypothesized that genetic diversity would be positively correlated with mean annual temperature across datasets. For our proxies of population size, we hypothesized that SVL should be negatively correlated with genetic diversity while range size and number of museum records should be positively correlated with genetic diversity. For ddRAD loci, we expected to find no influence of our proxies for direct environmentally driven selection (range of precipitation in the wettest and driest quarters, temperature seasonality), while we expected to see a positive relationship between these variables and genetic diversity for transcriptomic loci because environmental heterogeneity leads to selection for different alleles across a species range. Finally, we expected to see a negative relationship between genome size and genetic diversity, as larger genomes should have a lower recombination rate per bp and thus stronger linked selection, reducing diversity at linked sites. We summarize the hypothesized relationships between our predictor variables and genetic diversity in Figure 2.

We performed model testing separately for three genetic loci/species subsets: (A) heterozygous ddRAD loci, (B) heterozygous transcriptomic loci, and (C) ddRAD and transcriptomic loci for species in common between the two data types (hereafter referred to as the combined dataset). Because we expect most ddRAD loci to be noncoding, and thus less likely to be under direct selection, we compared the results of ddRAD and transcriptomic loci to understand the effects of direct selection on genetic diversity for the two locus types. Possible bias resulting from different species between the two full datasets was tested using the combined dataset. Eight of the species in the ddRAD dataset (*Bolitoglossa alberchi*, *B. nympha*, *B. odonnelli*, *B. xibalba*, *Bradytriton silus*, *Chiropterotriton mosaueri*, *Cryptotriton monzoni*, *and Oedipina uniformis*) do not have available genome size estimates and were excluded from initial analyses. Because we did not find a significant effect of genome size on genetic diversity for any subset (see Section 3), we reran regression analyses excluding genome size for this subset in order to expand the number of species included in our analysis.

We used both simple linear regression models assuming phylogenetic independence of all data points and stepwise phylogenetic linear regression to test for significant associations between predictor variables and genetic diversity. For simple linear models, we used the generic function lm in R v. 4.1.2. For stepwise phylogenetic regression, we used the package *phylolm* (Ho et al., 2016) and ran the stepwise models in both directions under a lambda model (bounded from 0 to 1) and an alpha of 0.05 to retain a variable in the model. We log‐transformed all variables prior to analysis and then used the scale function in R to center and scale the data. For phylogenetic stepwise analysis, we used the bolitoglossine phylogeny from Decena‐Segarra et al. (2020) pruned to include only species in our dataset. For all subsets, we used the Akaike Information Criterion (AIC) for model selection and the AICc weight as a measure of the proportion of the total amount of predictive power of the full set of models contained in each model being assessed. Finally, we estimated the summed AIC weight for each variable to quantify its importance across models.

## 3 RESULTS

We generated ddRAD sequence data for 56 species, with mean coverage per locus varying from 5 to 20× and the number of polymorphic loci varying from 2563 to 114,527 (Appendix 3) across libraries. For the 25 species for which we generated transcriptomic data, mean coverage by library varied from 23× to 62× across libraries and the number of polymorphic loci varied from 1097 to 5692. Nineteen libraries had both ddRAD and transcriptomic data. Correlation analyses revealed a significant negative relationship between mean coverage with genome size and the number of species included in a pool (Table 1), with the pool size explaining a substantial portion of the variance in coverage. The number of polymorphic ddRAD loci obtained was positively correlated with mean coverage.

**TABLE 1:** Correlation between mean coverage (coverage) and number of polymorphic loci (loci), number of species in the pool (pool size), and genome size (GS), as well as between genome size and polymorphic loci in ddRAD libraries.

Correlation	Estimate	*p* Value	Adjusted *R* ^2^
coverage ~ pool size	−0.61	1.25e–07	.40
coverage ~ GS	−0.47	.03	.08
loci ~ coverage	1.69	2.8e–04	.20
loci ~ GS	−0.19	.58	−.02

**FIGURE 3:**
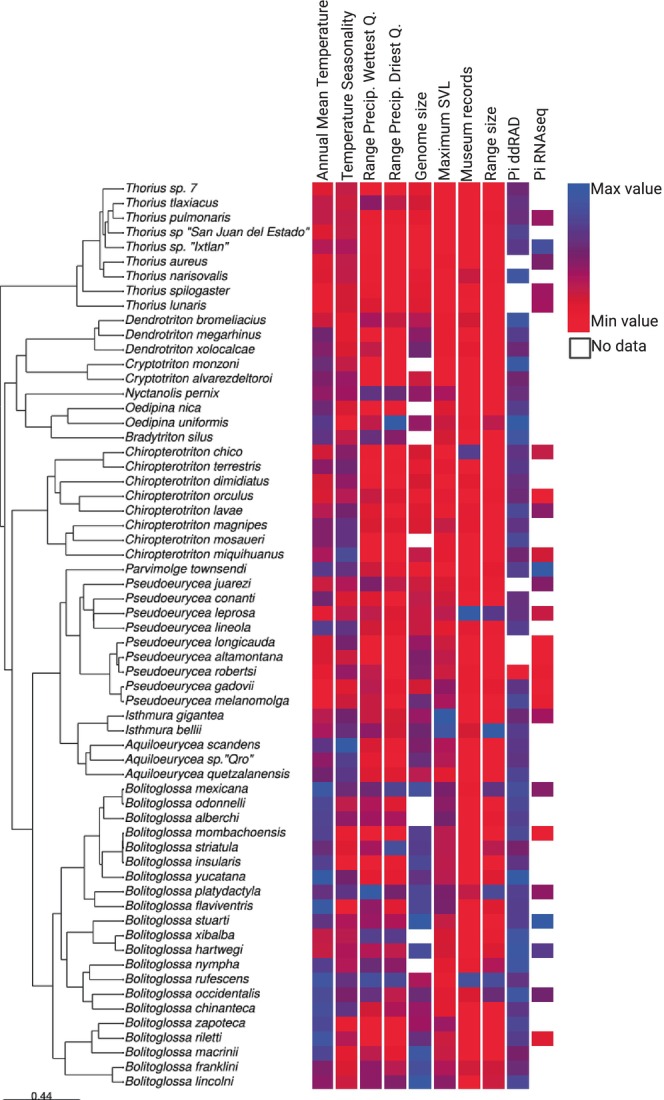
Phylogeny of bolitoglossine salamander species included in the study, with relative values of predictors and genetic diversity. For temperature seasonality and mean annual temperature, average values are shown when different vouchers were used for ddRAD and RNAseq datasets. Created with BioRender.com.

Estimates of genetic diversity of bolitoglossine salamander species ranged from pi = .0012–.015 for ddRAD loci and pi = .0020–.0072 for heterozygous transcriptomic loci (Figure 3, Appendix 4). For ddRAD loci, *Bolitoglossa yucatana* and *Pseudoeurycea robersti* had the highest and lowest genetic diversity, respectively. For heterozygous transcriptomic loci, *Bolitoglossa stuarti* had the highest diversity, while *Pseudoeurycea longicauda* had the lowest diversity. We did not find a significant correlation in genetic diversity between two types of loci (adjusted *R* ^2^ = .08, *p* = .13).

For ddRAD loci, no predictor variable was significantly associated with genetic diversity using a simple linear model or a phylogenetic stepwise model in either the full or the combined dataset. This remained true when the analyses were rerun excluding genome size to include the eight species without *C*‐value estimates.

For heterozygous transcriptomic loci, two proxies for environmental heterogeneity (range of precipitation of wettest and driest quarters) and one proxy of effective population size (SVL) are significant predictors of genetic diversity (Adjusted *R* ^2^ = .49; Table 2). Precipitation in the driest quarter is positively associated with genetic diversity, and the other two variables were negatively associated with genetic diversity. For heterozygous transcriptomic loci in the combined dataset, temperature seasonality and range of precipitation in the driest quarter were positively associated with genetic diversity and SVL, range of precipitation in the wettest quarter, and range size were negatively associated with genetic diversity (Adjusted; *R* ^2^ = .60). Results from ordinary least squares regression were broadly similar to those from phylostep although fewer variables were significant for the combined dataset (Table 3).

**TABLE 2:** Estimates of regression coefficients and *p* values from phylogenetic stepwise linear regression for transcriptomic loci.

Predictor	Heterozygous coding		Heterozygous coding (combined dataset)	
	Estimate	*p* Value	Estimate	*p* Value
Annual mean temp.	—	—	—	—
Temp. seasonality	—	—	0.00044	.037
Range precip. wettest *Q*	−0.0022	.0040	−0.0020	.012
Range precip. driest *Q*	0.0032	.0012	0.0032	.0018
SVL	−0.00079	.0066	−0.00091	.0065
Museum records	—	—	—	—
Range size	—	—	−0.00095	.023
Genome size	—	—	—	—
Lambda	0.68		1.0	
Adjusted *R* ^2^	.49		.60	

**TABLE 3:** Estimates of regression coefficients and *p* values from ordinary least squares regression for transcriptomic loci.

Predictor	Heterozygous coding		Heterozygous coding (combined dataset)	
	Estimate	*p* value	Estimate	*p* value
Annual mean temperature	0.00011	.76	−0.000046	.92
Temperature seasonality	0.00025	.32	0.00035	.34
Range precipitation wettest quarter	−0.0024	.026	−0.0024	.081
Range precipitation driest quarter	0.0035	.0039	0.0032	.044
SVL	−0.00090	.016	−0.00093	.058
Museum records	−0.00020	.70	−0.00028	.68
Range size	−0.00053	.35	−0.00031	.70
Genome size	0.00026	.48	0.00040	.42
Adjusted *R* ^2^	.51		.36	

The summed AIC weight for each variable reveals that body size (SVL) is the predictor with the most weight for genetic diversity of coding loci, with the range of precipitation in the driest quarter having the second highest summed AIC weight (Table 4). For ddRAD loci, annual mean temperature had the highest summed AIC weight although it was not a significant predictor of genetic diversity in phylogenetic stepwise linear models. Across subsets, genome size, range size, and number of museum records had consistently low summed AIC weights.

**TABLE 4:** Total AICw of each predictor by dataset.

Predictor	Total AICw				
	ddRAD		Heterozygous coding	Combined	
	With GS	Without GS		ddRAD	Coding
Annual mean temp.	0.58	0.69	0.35	0.44	0.24
Temp. seasonality	0.30	0.31	0.45	0.24	0.57
Range precip. wettest *Q*	0.35	0.53	0.60	0.30	0.47
Range precip. driest *Q*	0.38	0.49	0.68	0.35	0.69
SVL	0.24	0.26	0.74	0.20	0.72
Museum records	0.28	0.26	0.27	0.20	0.21
Range size	0.35	0.33	0.28	0.23	0.41
Genome size	0.26	—	0.22	0.24	0.16

## 4 DISCUSSION

We found contrasting results for predictors of genetic diversity for presumably noncoding (ddRAD) and coding loci. For ddRAD loci, no variable was significantly associated with genetic diversity for the full dataset or the set of species in both locus types. For transcriptomic loci, measures of environmental heterogeneity and proxies of population size explained a substantial portion of the variance in genetic diversity across both subsets. These results indicate that genetic diversity of noncoding regions of the genome may be unpredictable at shallower phylogenetic scales, in accordance with the results of Singhal et al. (2017), while diversity of coding loci may be more predictable.

Our estimates of genetic diversity from ddRAD loci (pi = 0.0012–0.015) vary by about one order of magnitude and fall within the range of those from previous studies that examined genetic diversity of eukaryotes using intergenic regions, introns and fourfold degenerate sites of pi = 0.0001–0.25 (Dutoit et al., 2017; Leffler et al., 2012; Mackintosh et al., 2019; Martin et al., 2016; Singhal et al., 2017). Singhal et al.'s (2017) estimates from single individuals (pi = 0.000214–0.00399) are lower but, like our results, vary by approximately one order of magnitude. Compared to other ddRAD studies of salamander genetic diversity, our estimates are almost 10 times lower than those for *Necturus maculosus* (Proteidae, pi = 0.103–0.145) but higher than for *Ambystoma opacum* and *A. talpoideum* (Ambystomatidae, pi = 7.46e‐15–2.51e‐07) (Murphy et al., 2018; Nunziata et al., 2017). For transcriptomic loci, our estimates (pi = 0.0020–0.0072) are lower than those for multiple species of European butterflies (pi = 0.0044–0.428) estimated from fourfold degenerate sites (Mackintosh et al., 2019) and those from 76 metazoans estimated from synonymous sites (Romiguier et al., 2014); this difference is likely explained at least in part by the fact that we used all sites, some of which are likely to be under direct selection. Thus, our results are broadly comparable to levels of genetic diversity estimated in previous studies.

The lack of an association between any proxies of mutation rate or population size and genetic diversity for our ddRAD datasets is somewhat surprising because these relationships are a central prediction of the neutral theory. Salamanders have been shown to have robust DNA damage repair mechanisms (Sousounis et al., 2020), and lower DNA deletion rates in salamanders compared to other vertebrates could be explained by higher fidelity of double‐stranded break repair (Sun, Shepard, et al., 2012). High‐fidelity DNA damage repair, together with relatively low metabolic rates of salamanders in general, could explain why we found no effect of mutation rate on genetic diversity for ddRAD or transcriptomic loci. In a larger ddRAD dataset at a somewhat shallower phylogenetic scale, Singhal et al. (2017) found that the number of museum records, range size, and historical stability of species distributions predicted some variation in species‐wide genetic diversity, while only the number of museum records predicted within‐population diversity; they did not include proxies of mutation rate or linked selection in their analyses. None of their models explained a large proportion of the variance in genetic diversity across populations or species. Thus, our results are broadly in agreement in that genetic diversity of noncoding loci appears to be mostly unpredictable at shallower phylogenetic scales, but the disagreement between our results and basic population genetic predictions remains unexplained.

All proxies for population size used in our study relate to the number of individuals in a population (census population size, *N*_c_) rather than to effective population size (*N*_e_), a measure of the strength of genetic drift in a population. Although *N*_c_ and *N*_e_ should be correlated (although not equal) under demographic equilibrium conditions, Singhal et al. (2017) discussed how changes in historical demography (population bottlenecks and expansions) can break down the correlation between *N*_c_ and *N*_e_. Because plethodontid salamanders are intimately tied to specific habitats with sufficient moisture, including cloud forests whose distribution is predicted to have been dynamic under past climatic conditions (Guevara, 2020; Ramírez‐Barahona & Eguiarte, 2013), many species are likely to have experienced substantial range expansions and contractions over time, with corresponding changes in population size. Although we did not attempt to quantify historical range stability in our analysis, historical demography could be one explanation for the lack of an observed effect of proxies for population size on genetic diversity of noncoding loci.

The difference between our results and those of Singhal et al. (2017) could be either due to differences in the study system (one genus of lizards [*Ctenotus*] vs. several related genera of salamanders) or because of differences in methodology and data quality. Likely as a result of the large genome size of all salamanders in this study, our depth of coverage for all ddRAD loci (mean coverage 5×–20× per locus across libraries) was low compared to mean depth of coverage for transcriptomic loci (23×–62×). This, combined with our use of only one individual per species, means that we were unlikely to uncover low‐frequency variants within species. The significant correlation between mean coverage and genome size (Table 1) suggests that lower coverage may have impeded our ability to call SNPs in species with larger genomes, although we found only a marginally significant correlation between the number of polymorphic ddRAD loci and genome size. This issue means that our values of genetic diversity for ddRAD loci are likely underestimated (perhaps substantially so), particularly in species with larger genomes, although our use of only heterozygous ddRAD loci should partially overcome this issue. Downwardly biased estimates of genetic diversity for ddRAD loci may not have allowed us to uncover associations between proxies of population size and genetic diversity, potentially explaining the discrepancy between our results and those for *Ctenotus*.

Our proxy for genome‐wide recombination rate (genome size) was not a significant predictor of genetic diversity estimated for either data type. This could either mean that linked selection does not affect genetic diversity strongly in this group, contrary to the results of Corbett‐Detig et al. (2015), or that genome size is a poor proxy for genome‐wide recombination rate. Although data on the number of chiasmata that occur during meiosis are lacking for species in our study, the number of chiasmata may increase with genome size in this group, in contrast to mammals and birds where it appears to be roughly constant per chromosome arm (Macgregor, 1993; Morescalchi & Galgano, 1973; Pardo‐Manuel de Villena & Sapienza, 2001). If the number of chiasmata increases approximately linearly with genome size in bolitoglossine salamanders, then genome size would not be expected to be associated with recombination rate per bp. Thus, while finding a negative association between genome size and genetic diversity would have supported the hypothesis that lower recombination rates in larger genomes result in lower genetic diversity because of background selection, the lack of such an association does not necessarily mean that linked selection is unimportant as a determinant of genetic diversity. Linkage maps and reference genomes for the species in this study would be necessary to determine if a correlation exists between the number of chiasmata during meiosis and genome size for bolitoglossines.

In contrast to the results for ddRAD loci, the best model for genetic diversity of heterozygous transcriptomic loci shows significant associations of proxies of environmental heterogeneity and population size with genetic diversity. Population size is typically negatively associated with body size, as larger organisms tend to have lower average population density (White et al., 2007). The negative association between SVL and genetic diversity thus conforms to the predictions under the neutral theory. Range size should be positively correlated with population size, and thus genetic diversity, but we instead found a negative relationship in the combined dataset. Of these two variables, SVL has a much higher AIC weight for transcriptomic loci (Table 4), indicating its importance across a broader set of models. For environmental heterogeneity, however, results are mixed; the range of precipitation of the driest quarter (both transcriptomic datasets) and temperature seasonality (combined dataset only) are positively associated with genetic diversity (as predicted) while the range of precipitation of the wettest quarter is negatively associated with genetic diversity. The summed AIC weight for these three variables is high (Table 4), indicating that they are important in a broad range of better models. Salamanders require moist microhabitats to survive, and moisture constraints during the drier months may be a stronger source of selection compared to conditions in the wettest quarter of the year. The results for environmental heterogeneity measures rely on the assumption that variation in conditions across a species range should lead to higher genetic diversity as a result of selection for different variants across space. The inclusion of more than one individual per species would provide a more direct test of this hypothesis.

The large difference in explanatory power and predictors between ddRAD and transcriptomic loci cannot be due to species composition alone, as the combined dataset had no predictor significantly associated with ddRAD genetic diversity while the model for transcriptomic genetic diversity had five predictors and much higher explanatory power (*R* ^2^ = .59). Our transcriptomic data have substantially higher coverage depth compared to the ddRAD loci, and thus estimates of genetic diversity are likely more accurate for transcriptomic data than for ddRAD data.

Despite the relatively shallow phylogenetic scale of our study, we found support for effects of proxies of population size and environmental heterogeneity on genetic diversity of coding loci, largely in accordance with the predictions of basic population genetic theory. The lack of any significant association with genetic diversity for ddRAD loci may be either because of methodological issues or because neutral diversity is simply mostly unpredictable at shallow phylogenetic scales. Diversity of noncoding loci (such as microsatellites) is often used as a proxy for adaptive genetic diversity, which should determine the ability of species to adapt to environmental changes such as climatic warming. The discrepancy between results from our two datasets may mean that, while genetic diversity estimates from noncoding loci are increasingly incorporated in conservation and management strategies, it may be more appropriate to use diversity estimates from coding loci, upon which selection is more likely to act directly. As an alternative strategy, conservation efforts could attempt to protect populations that are likely to maximize genetic variation based on the associations we found. For salamanders, our results suggest that larger species in environments with less variation in dry season precipitation are likely to have the lowest genetic diversity, and thus might struggle to adapt to environmental changes brought by climate change and anthropogenic disturbance. High‐quality whole‐genome data for closely related species, together with linkage maps or recombination estimates across the genome, will provide a stronger test of the impact of mutation, selection, and drift on genetic diversity of wild populations.

## AUTHOR CONTRIBUTIONS

**María Guadalupe Segovia‐Ramírez:** Conceptualization (equal); data curation (lead); formal analysis (equal); investigation (lead); methodology (lead); writing – original draft (equal). **Obed Ramírez‐Sánchez:** Formal analysis (equal); methodology (supporting); writing – review and editing (supporting). **Louis Paul Decena Segarra:** Data curation (supporting); investigation (supporting); writing – review and editing (supporting). **Hairo Rios‐Carlos:** Data curation (supporting); investigation (supporting); writing – review and editing (supporting). **Sean M. Rovito:** Conceptualization (equal); funding acquisition (lead); investigation (supporting); project administration (lead); writing – original draft (equal).

## CONFLICT OF INTEREST STATEMENT

The authors have no conflict of interest to declare.

## Data Availability

Illumina sequence data for transcriptomes and ddRAD sequences are deposited in the NCBI Short Read Archive (BioProject PRJNA1023252). Specimen locality data used to estimate climatic variability, range size, and number of museums are deposited in Zenodo (http://doi.org/10.5281/zenodo.8397163).

## APPENDIX 1

### 1.1

Specimens included in ddRAD and RNAseq datasets. IBH, Instituto de Biología, UNAM; MVZ, Museum of Vertebrate Zoology, University of California, Berkeley; SMR, Uncatalogued specimens collected by Sean Michael Rovito; UCR, Museo de Zoología, Universidad de Costa Rica.

**Table:**

Species	Voucher ddRAD	Voucher RNAseq
*Aquiloeurycea quetzalanensis*	IBH 32125	NA
*Aquiloeurycea scandens*	IBH 33585	NA
*Aquiloeurycea* sp. Qro	IBH 32119	NA
*Bolitoglossa alberchi*	MVZ 272289	NA
*Bolitoglossa chinanteca*	IBH 32981	NA
*Bolitoglossa flaviventris*	IBH 32974	NA
*Bolitoglossa franklini*	IBH 32968	NA
*Bolitoglossa hartwegi*	IBH 32081	IBH 32081
*Bolitoglossa insularis*	IBH 32967	NA
*Bolitoglossa lincolni*	MVZ 269573	NA
*Bolitoglossa macrinii*	IBH 32969	NA
*Bolitoglossa mexicana*	IBH 32184	IBH 32184
*Bolitoglossa mombachoensis*	IBH 32902	IBH 32967
*Bolitoglossa nympha*	MVZ 269890	NA
*Bolitoglossa occidentalis*	IBH 32982	IBH 32982
*Bolitoglossa odonnelli*	MVZ 263498	NA
*Bolitoglossa platydactyla*	IBH 32929	IBH 32913
*Bolitoglossa riletti*	IBH 32076	IBH 32076
*Bolitoglossa rufescens*	IBH 33024	NA
*Bolitoglossa striatula*	IBH 33071	NA
*Bolitoglossa stuarti*	IBH 33000	IBH 33000
*Bolitoglossa xibalba*	MVZ 265604	NA
*Bolitoglossa yucatana*	SMR3434	SMR3507
*Bolitoglossa zapoteca*	SMR3465	NA
*Bradytriton silus*	MVZ 263971	NA
*Chiropterotriton chico*	IBH 32124	IBH
*Chiropterotriton dimidiatus*	IBH 32173	NA
*Chiropterotriton lavae*	IBH 32915	IBH 32915
*Chiropterotriton magnipes*	IBH 33144	NA
*Chiropterotriton miquihuanus*	IBH 33119	IBH 33119
*Chiropterotriton mosaueri*	IBH 33131	NA
*Chiropterotriton orculus*	IBH 33318	IBH 33318
*Chiropterotriton terrestris*	IBH 32955	NA
*Cryptotriton alvarezdeltoroi*	IBH 29568	NA
*Cryptotriton monzoni*	MVZ 265530	NA
*Dendrotriton bromeliacius*	MVZ 263576	NA
*Dendrotriton megarhinus*	IBH 33305	NA
*Dendrotriton xolocalcae*	IBH 33298	NA
*Isthmura bellii*	IBH 33010	NA
*Isthmura gigantea*	IBH 32306	IBH 32303
*Nyctanolis pernix*	IBH 33299	NA
*Oedipina nica*	MVZ 263774	NA
*Oedipina uniformis*	UCR 20982	NA
*Parvimolge townsendi*	SMR2923	SMR3171
*Pseudoeurycea altamontana*	NA	IBH 32204
*Pseudoeurycea conanti*	IBH 32237	NA
*Pseudoeurycea gadovii*	IBH 32300	IBH 32262
*Pseudoeurycea juarezi*	NA	IBH 32426
*Pseudoeurycea leprosa*	IBH 32225	IBH 32315
*Pseudoeurycea lineola*	IBH 32329	NA
*Pseudoeurycea longicauda*	NA	IBH 32205
*Pseudoeurycea melanomolga*	IBH 32275	IBH 32214
*Pseudoeurycea robertsi*	IBH 32356	IBH 32356
*Thorius aureus*	NA	IBH 32451
*Thorius* sp. “Ixtlan”	IBH 32478	IBH 33642
*Thorius lunaris*	NA	IBH 32132
*Thorius narisovalis*	IBH 32143	NA
*Thorius pulmonaris*	IBH 32158	NA
*Thorius* sp. “San Juan del Estado”	SMR2593	NA
*Thorius* sp. 7	IBH 32169	NA
*Thorius spilogaster*	NA	IBH 32171
*Thorius tlaxiacus*	IBH 32145	NA

## APPENDIX 2

### 2.1

Values of all predictor variables used for each species in regression analyses.

**Table:**

Species	Annual mean temperature (°C) ddRAD/RNAseq	Temperature seasonality in % ddRAD/RNAseq	Precipitation in the wettest quarter range (mm)	Precipitation in the driest quarter range (mm)	Genome size (pg)	Maximum SVL (mm)	Museum records	Distribution range (km^2^)
*Aquiloeurycea quetzalanensis*	17.9	226.8	410.0	100	35.3	44.8	16	476.091
*Aquiloeurycea scandens*	19.9	303.4	448.0	45	49.5	71	358	802.5
*Aquiloeurycea* sp. Qro	16.1	236.6	285	19	57.2	68.6	10	270.7
*Bolitoglossa alberchi*	21.5	166.5	883.0	217	NA	97.1	196	1306.1
*Bolitoglossa chinanteca*	23	218.6	506.0	209	43.8	44	14	256.4
*Bolitoglossa flaviventris*	25.2	60.5	978.0	95	70.4	95	337	1875.4
*Bolitoglossa franklini*	16.8	107.2	999.0	188	74.2	71.8	1742	2616.3
*Bolitoglossa hartwegi*	12.9	145.7	750	174	72.7	53.6	243	2075.2
*Bolitoglossa insularis*	21.3	71.1	59	56	70.9	64.7	6	123.4
*Bolitoglossa lincolni*	16.1	121.1	1002.0	290	83.4	86	504	2766.5
*Bolitoglossa macrinii*	21.9	97.1	660.0	80	81.1	59.1	118	730.6
*Bolitoglossa mexicana*	25	201.7	1265.0	444	76.3	93.3	659	6995.3
*Bolitoglossa mombachoensis*	21.3	83.3	85	9	66.9	66	58	195.7
*Bolitoglossa nympha*	21	142.4	1311.0	270	NA	43.2	756	3769.2
*Bolitoglossa occidentalis*	22.4	184.8	1374	173	56.1	45.6	1455	6367.1
*Bolitoglossa odonnelli*	22	130.7	792.0	98	NA	86	85	450.8
*Bolitoglossa platydactyla*	17.5/20.9	219.7/221.5	2232	320	70.3	93.0	2096	8579.1
*Bolitoglossa riletti*	24.1	140.9	100	24	57.9	57.8	69	499.2
*Bolitoglossa rufescens*	23.9	212.7	1872	469	39.5	37.5	6313	8722.4
*Bolitoglossa striatula*	18.8	101.6	852	494	65.8	66	257	3433.7
*Bolitoglossa stuarti*	19.7	145.3	926	208	87.2	60	48	730.4
*Bolitoglossa xibalba*	13	136.4	1621	418	NA	52.7	74	1200.5
*Bolitoglossa yucatana*	26.1	190.7	274	72	60.1	66	113	1940
*Bolitoglossa zapoteca*	22.2	83.9	25	1	42.2	80.5	14	124.1
*Bradytriton silus*	19.8	130	1354	280	NA	53.3	37	420
*Chiropterotriton chico*	12	174.6	178	19	28	23.8	5417	377.1
*Chiropterotriton dimidiatus*	11.9	166.4	176	20	27.8	27.0	1156	344.4
*Chiropterotriton lavae*	13.8	191.9	368	46	25.8	34.9	1035	362.6
*Chiropterotriton magnipes*	16.9	215.5	422	99	27	60	890	729
*Chiropterotriton miquihuanus*	14.6	254.8	34	4	33.3	40.9	37	203
*Chiropterotriton mosaueri*	16.9	215.5	0.001	0.001	NA	42.9	19	95.8
*Chiropterotriton orculus*	11.6	139.6	576	109	18.9	43	193	1263.6
*Chiropterotriton terrestris*	16.2	196.1	147	43	21.9	43	429	342.4
*Cryptotriton alvarezdeltoroi*	17	162.4	36	12	30.7	26.6	6	143.5
*Cryptotriton monzoni*	18.7	127.9	0.001	0.001	NA	23.2	6	93.5
*Dendrotriton bromeliacius*	12.3	96.6	847	152	36.9	37.9	1667	246.9
*Dendrotriton megarhinus*	18	99.1	20	9	46.6	37.5	258	124
*Dendrotriton xolocalcae*	16.8	107.2	618	15	54.5	37.2	885	303
*Isthmura bellii*	14.7	205.9	1004	134	54	146	1635	10770.6
*Isthmura gigantea*	13.6	193.6	577	128	43.8	161	102	863.7
*Nyctanolis pernix*	15.9	157.2	1436	349	45.1	73.6	35	596.1
*Oedipina nica*	18.4	110.3	108	48	NA	48.5	27	401.7
*Oedipina uniformis*	20.7	65.6	638	593	44.9	58.3	674	3252.1
*Parvimolge townsendi*	20.6	214.5	533	84	20.6	25.6	515	1398.1
*Pseudoeurycea altamontana*	11.6	120	145	16	49.6	62.6	137	766.2
*Pseudoeurycea conanti*	18.1	109.2	400	4	2.2	56	13	265.7
*Pseudoeurycea gadovii*	8/9.6	86.7/114.1	696	80	28.9	83.9	847	1417.7
*Pseudoeurycea juarezi*	12.5	144.1	1127	171	30.7	51.3	742	1120.1
*Pseudoeurycea leprosa*	8.6/12.3	127.8/137.7	639	109	32	65.0	7515	7572.1
*Pseudoeurycea lineola*	21	216.3	505	93	31.6	44.0	1059	885.4
*Pseudoeurycea longicauda*	11.4	189.3	115	16	44.1	56.7	193	668.1
*Pseudoeurycea melanomolga*	7.3/8.6	109.9/127.8	576	44	56.8	73.5	204	563.3
*Pseudoeurycea robertsi*	10.4	165.7	650	32	48.9	57.8	814	811.7
*Thorius aureus*	11.2	130.1	163	26	16.7	34.9	124	141.7
*Thorius* sp. “Ixtlan”	13.8/14.1	145/149	0.001	0.001	15.1	25.3	17	92.4
*Thorius lunaris*	9.6	114.1	412	41	24.9	31.9	435	334.2
*Thorius narisovalis*	11.3	129.2	162	20	22.9	31.1	1919	872.1
*Thorius pulmonaris*	13.1	127.3	165	57	22.2	27.6	489	1028.7
*Thorius* sp. “San Juan del Estado”	11.2	126.4	28	12	12.4	29.3	11	148.1
*Thorius* sp. 7	11	126.5	41	15	23.6	25.7	20	162.5
*Thorius spilogaster*	9.6	114.1	223	30	23.5	26.7	606	293.1
*Thorius tlaxiacus*	13.1	120.0	1004	168	28.9	31.0	158	470.3

## APPENDIX 3

### 3.1

Number of polymorphic loci from ddRAD and RNaseq libraries.

**Table:**

Species	ddRAD	Transcriptomes
*Aquiloeurycea quetzalanensis*	19,187	NA
*Aquiloeurycea scandens*	12,364	NA
*Aquiloeurycea* sp. Qro	13,870	NA
*Bolitoglossa alberchi*	5978	NA
*Bolitoglossa chinanteca*	14,392	NA
*Bolitoglossa flaviventris*	9785	NA
*Bolitoglossa franklini*	114,527	NA
*Bolitoglossa hartwegi*	4382	3239
*Bolitoglossa insularis*	22,446	NA
*Bolitoglossa lincolni*	9781	NA
*Bolitoglossa macrinii*	82,464	NA
*Bolitoglossa mexicana*	3442	2348
*Bolitoglossa mombachoensis*	6677	1097
*Bolitoglossa nympha*	6131	NA
*Bolitoglossa occidentalis*	4467	2070
*Bolitoglossa odonnelli*	6964	NA
*Bolitoglossa platydactyla*	14,125	2100
*Bolitoglossa riletti*	18,484	2923
*Bolitoglossa rufescens*	82,073	NA
*Bolitoglossa striatula*	75,258	NA
*Bolitoglossa stuarti*	75,361	4223
*Bolitoglossa xibalba*	12,773	NA
*Bolitoglossa yucatana*	5237	NA
*Bolitoglossa zapoteca*	3043	NA
*Bradytriton silus*	6598	NA
*Chiropterotriton chico*	40,644	2329
*Chiropterotriton dimidiatus*	50,902	NA
*Chiropterotriton lavae*	17,895	2874
*Chiropterotriton magnipes*	24,040	NA
*Chiropterotriton miquihuanus*	37,906	2050
*Chiropterotriton mosaueri*	13,771	NA
*Chiropterotriton orculus*	25,440	2279
*Chiropterotriton terrestris*	20,897	NA
*Cryptotriton alvarezdeltoroi*	42,586	NA
*Cryptotriton monzoni*	2563	NA
*Dendrotriton bromeliacius*	2173	NA
*Dendrotriton megarhinus*	23,424	NA
*Dendrotriton xolocalcae*	27,132	NA
*Isthmura bellii*	22,514	NA
*Isthmura gigantea*	41,431	3703
*Nyctanolis pernix*	18,067	NA
*Oedipina nica*	16,555	NA
*Oedipina uniformis*	3395	NA
*Parvimolge townsendi*	41,793	5692
*Pseudoeurycea altamontana*	NA	3116
*Pseudoeurycea conanti*	23,579	NA
*Pseudoeurycea gadovii*	29,429	4434
*Pseudoeurycea juarezi*	NA	4155
*Pseudoeurycea leprosa*	35,360	4693
*Pseudoeurycea lineola*	40,004	NA
*Pseudoeurycea longicauda*	NA	3507
*Pseudoeurycea melanomolga*	12,171	3314
*Pseudoeurycea robertsi*	6956	3312
*Thorius aureus*	NA	2318
*Thorius* sp. *Ixtlan*	21,623	2211
*Thorius* sp. *lunaris*	NA	2824
*Thorius narisovalis*	3477	NA
*Thorius pulmonaris*	62,847	2974
*Thorius* sp. “San Juan del Estado”	31,532	NA
*Thorius* sp. 7	42,532	NA
*Thorius spilogaster*	NA	3340
*Thorius tlaxiacus*	53,669	NA

## APPENDIX 4

### 4.1

Nucleotide diversity estimation for heterozygous ddRAD and heterozygous and all transcriptomic loci per species.

**Table:**

Species	ddRAD	Heterozygous coding	All coding
*Aquiloeurycea quetzalanensis*	0.012	NA	NA
*Aquiloeurycea scandens*	0.011	NA	NA
*Aquiloeurycea* sp. “Qro”	0.010	NA	NA
*Bolitoglossa alberchi*	0.011	NA	NA
*Bolitoglossa chinanteca*	0.011	NA	NA
*Bolitoglossa flaviventris*	0.011	NA	NA
*Bolitoglossa franklini*	0.0093	NA	NA
*Bolitoglossa hartwegi*	0.014	0.0057	0.0042
*Bolitoglossa insularis*	0.010	NA	NA
*Bolitoglossa lincolni*	0.012	NA	NA
*Bolitoglossa macrinii*	0.0084	NA	NA
*Bolitoglossa mexicana*	0.013	0.0045	0.0032
*Bolitoglossa mombachoensis*	0.012	0.0024	0.00083
*Bolitoglossa nympha*	0.014	NA	NA
*Bolitoglossa occidentalis*	0.014	0.0049	0.0029
*Bolitoglossa odonnelli*	0.012	NA	NA
*Bolitoglossa platydactyla*	0.011	0.0043	0.0026
*Bolitoglossa riletti*	0.011	0.0029	0.0015
*Bolitoglossa rufescens*	0.010	NA	NA
*Bolitoglossa striatula*	0.0084	NA	NA
*Bolitoglossa stuarti*	0.011	0.0072	0.0056
*Bolitoglossa xibalba*	0.013	NA	NA
*Bolitoglossa yucatana*	0.015	NA	NA
*Bolitoglossa zapoteca*	0.012	NA	NA
*Bradytriton silus*	0.013	NA	NA
*Chiropterotriton chico*	0.010	0.0034	0.0017
*Chiropterotriton dimidiatus*	0.0096	NA	NA
*Chiropterotriton lavae*	0.012	0.0044	0.0024
*Chiropterotriton magnipes*	0.010	NA	NA
*Chiropterotriton miquihuanus*	0.0089	0.0032	0.0014
*Chiropterotriton mosaueri*	0.012	NA	NA
*Chiropterotriton orculus*	0.0092	0.0024	0.00099
*Chiropterotriton terrestris*	0.011	NA	NA
*Cryptotriton alvarezdeltoroi*	0.0088	NA	NA
*Cryptotriton monzoni*	0.014	NA	NA
*Dendrotriton bromeliacius*	0.014	NA	NA
*Dendrotriton megarhinus*	0.011	NA	NA
*Dendrotriton xolocalcae*	0.0091	NA	NA
*Isthmura bellii*	0.011	NA	NA
*Isthmura gigantea*	0.0091	0.0041	0.0027
*Nyctanolis pernix*	0.0095	NA	NA
*Oedipina nica*	0.011	NA	NA
*Oedipina uniformis*	0.015	NA	NA
*Parvimolge townsendi*	0.011	0.0071	0.00595
*Pseudoeurycea altamontana*	NA	0.00204	0.0011
*Pseudoeurycea conanti*	0.0097	NA	NA
*Pseudoeurycea gadovii*	0.010	0.0024	0.0015
*Pseudoeurycea juarezi*	NA	0.0045	0.0033
*Pseudoeurycea leprosa*	0.0099	0.0034	0.0021
*Pseudoeurycea lineola*	0.011	NA	NA
*Pseudoeurycea longicauda*	NA	0.00203	0.00103
*Pseudoeurycea melanomolga*	0.012	0.0025	0.0011
*Pseudoeurycea robertsi*	0.0012	0.0025	0.0012
*Thorius aureus*	NA	0.0047	0.0023
*Thorius boreas*	NA	NA	NA
*Thorius* sp. “Ixtlan”	0.011	0.0064	0.0039
*Thorius lunaris*	NA	0.0041	0.0024
*Thorius narisovalis*	0.014	NA	NA
*Thorius pulmonaris*	0.0095	0.0042	0.0027
*Thorius* sp. “San Juan del Estado”	0.012	NA	NA
*Thorius* sp. 7	0.0091	NA	NA
*Thorius spilogaster*	NA	0.0041	0.0025
*Thorius tlaxiacus*	0.0099	NA	NA
